# Response to Questionable assumptions mar modelling of Kenya home‐based testing campaigns ‐ a comment on “Optimal timing of HIV home‐based counselling and testing rounds in Western Kenya” (Olney et al. 2018)

**DOI:** 10.1002/jia2.25231

**Published:** 2019-01-22

**Authors:** Jack J Olney, Jeffrey W Eaton, Paula Braitstein, Joseph W Hogan, Timothy B Hallett

**Affiliations:** ^1^ Centre for Health Economics & Policy Innovation Imperial College Business School London UK; ^2^ Department of Infectious Disease Epidemiology Imperial College London London UK; ^3^ Dalla Lana School of Public Health University of Toronto Toronto ON Canada; ^4^ Department of Medicine College of Health Sciences School of Medicine Moi University Eldoret Kenya; ^5^ Department of Biostatistics and Center for Statistical Sciences Brown University School of Public Health Providence RI USA

**Keywords:** modelling

We appreciate the letter in response to our article “Optimal timing of HIV home‐based counselling and testing rounds in Western Kenya 1.” The authors appraise the assumptions made by the model and question whether the modelled HBCT programme in western Kenya could be improved 2.

As the authors are aware, high prevalence settings often require active outreach to identify a meaningful proportion of the infected population 3. However, timely linkage to care following diagnosis remains a challenge 4. Several studies are looking at innovative means of addressing this, including the use of peer navigators 5, mHealth initiatives 6, and same‐day ART start 7.

Meanwhile, a different ‐ but still valid ‐ question concerns how best to use an existing programme, in this case through repeating it to test more people. The model is based on real data from AMPATH which arguably makes it conclusions better suited to policy than arguments based on hypothetical extrapolations. It was necessary to make assumptions, and these were not with the intention of being cautiously conservative about the impact of the programme.

Further model analyses could indeed compare the impact of different types of HBCT programme, as well as assessing ancillary benefits such as knowledge transfer to the community and NCD testing.

## Competing interests

The authors declare that they have no competing interests.

## Authors’ contributions

JJO and TBH drafted the initial version of the letter. JWE, PB and JWH reviewed and provided revisions to the draft prior, before JJO circulated the finalized letter for approval from all authors prior to submission.
